# Employee–AI Collaboration and Career Sustainability: The Role of Job Crafting and AI Job Role Clarity

**DOI:** 10.3390/bs16081451

**Published:** 2026-08-21

**Authors:** Huiling Jiang, Yuanyuan Zhang, Shumin Yan

**Affiliations:** School of Economics and Management, Tongji University, Shanghai 200092, China; helenejiang@tongji.edu.cn (H.J.);

**Keywords:** employee–AI collaboration, job crafting, AI job role clarity, career sustainability

## Abstract

Artificial intelligence (AI) is reshaping the global labor market and creating both challenges and opportunities for employees. This study conceptualizes AI as a collaborative partner in the workplace and examines how employee–AI collaboration relates to career sustainability. Drawing on conservation of resources theory and self-determination theory, the study proposes that job crafting mediates this relationship and that AI job role clarity strengthens the positive effect of employee–AI collaboration on job crafting. Three-wave survey data were collected from 398 employees who were required to use AI as part of regular work. The results showed that employee–AI collaboration was significantly associated with career sustainability, that job crafting mediated the relationship between employee–AI collaboration and career sustainability, and that AI job role clarity positively moderated the effect of employee–AI collaboration on job crafting. These findings extend research on AI adoption and job crafting by showing that employee–AI collaboration constitutes an interdependent work arrangement whose long-term career value depends on employees’ proactive work redesign and clear human–AI role boundaries. The findings further suggest that organizations can support employees’ proactive adaptation and career development by clarifying human–AI role boundaries, providing opportunities for skill development, and creating conditions that enable employees to redesign their work effectively.

## 1. Introduction

As AI becomes increasingly embedded in contemporary workplaces, employee–AI collaboration is emerging as an important work behavior that may enhance employees’ productivity, well-being, and long-term career development (Liang et al., 2025; Wu et al., 2024). Rather than functioning solely as a substitute for human labor, AI can also serve as a collaborative partner that reshapes how work is designed and performed (Chen & Zhao, 2025; Y. B. Li et al., 2025). Although AI was initially viewed primarily as a tool for automation and efficiency enhancement, recent research suggests that it should also be understood as a collaborative partner that augments human capabilities (Anthony et al., 2023; Chowdhury et al., 2022). Such employee–AI collaboration has been associated with improved work outcomes, including greater productivity and creativity, because it enables employees to combine human judgment with AI-enabled speed and analytical power (Jia et al., 2024; Kong et al., 2023). As AI becomes more deeply embedded in work, understanding how employees adapt to and benefit from this collaborative relationship has become an important issue in organizational research.

However, the popularity of AI has also raised concerns about employees’ long-term career development. Existing discussions on AI in the workplace have often emphasized job displacement, job insecurity, technostress, and the erosion of traditional skill boundaries (Brougham & Haar, 2018; J. Li & Huang, 2020). While such concerns are important, they do not fully capture the broader reality that many employees are not simply competing against AI, but are expected to collaborate with it. A key question is whether employee–AI collaboration can support career sustainability, that is, employees’ ability to maintain a viable, adaptable, and meaningful career over time (Chin et al., 2019; De Vos et al., 2020). This is especially important in an era of boundaryless and self-directed careers, in which long-term career success depends not only on stable employment, but also on adaptability, employability, developmental flexibility, and the capacity to renew career resources over time (Barthauer et al., 2020; Van der Heijden et al., 2020). Although recent work has begun to link AI-related factors to career outcomes, such as trust in AI and career sustainability (Kong et al., 2023), empirical evidence on whether and how employee–AI collaboration contributes to sustainable careers remains limited.

Although research on AI in the workplace has expanded rapidly, three important gaps remain. First, much of the existing literature conceptualizes AI primarily in terms of organizational adoption, employee acceptance, or frequency of use (Jia et al., 2024). These perspectives capture employees’ exposure to AI but do not fully represent employee–AI collaboration as an ongoing and interdependent work process in which employees coordinate with AI systems, incorporate AI-generated information into their decisions, and redistribute tasks between human and technological agents (Anthony et al., 2023; Sowa et al., 2021). This distinction is theoretically important because the adoption or use of AI does not necessarily indicate that employees have developed a collaborative work relationship with it or obtained enduring developmental value from that relationship. Second, prior research connecting AI-related changes with job crafting has primarily examined whether organizational AI adoption, employee appraisals of AI, or collaboration with service robots stimulates proactive work redesign (Cheng et al., 2023; He et al., 2024; Song et al., 2022). These studies have provided valuable evidence that technological change can alter how employees shape their jobs. However, they have paid less attention to the longer-term function of job crafting in converting AI-enabled work experiences into sustainable career resources. Job crafting may be more than a proximal employee response to AI (Akkermans & Tims, 2017; Wong et al., 2021). It may constitute the behavioral process through which employees transform the time, information, autonomy, and learning opportunities obtained from AI collaboration into adaptability, employability, developmental flexibility, and career renewal (Tims et al., 2012; Zhang & Parker, 2019). Third, research has begun to connect AI-related factors with career sustainability. For example, Kong et al. (2023) examined employee–AI collaboration within the relationship between trust in AI and career sustainability. Nevertheless, the employee-driven behavioral process through which collaboration with AI contributes to sustainable careers remains insufficiently understood. It is also unclear why similar levels of employee–AI collaboration may produce stronger proactive responses for some employees than for others (Chowdhury et al., 2022; Jarrahi, 2018). AI job role clarity may address this question because employees are more likely to redesign their work when they clearly understand what AI is expected to do, how human and AI responsibilities are divided, and how collaboration with AI fits their own work and career development (Brougham & Haar, 2018; J. Li & Huang, 2020). Accordingly, further research is needed to explain both how employee–AI collaboration is converted into sustainable career resources and when this conversion process becomes more effective.

To address these gaps, we conceptualize job crafting as a behavioral conversion mechanism linking employee–AI collaboration with career sustainability. Employee–AI collaboration may provide immediate task-related, informational, temporal, and motivational benefits, but these benefits do not automatically become lasting career resources. Through job crafting, employees can reinvest such benefits by modifying their tasks, seeking developmental challenges, strengthening work relationships, and redefining the meaning of their work. We further identify AI job role clarity as a coordination condition that determines how effectively employees can undertake this resource conversion. When the respective roles, responsibilities, and expectations of employees and AI are clear, employees are better able to translate collaborative experiences into proactive work redesign and, ultimately, more sustainable careers. To explain these relationships, we draw on conservation of resources theory and self-determination theory. Conservation of resources theory suggests that individuals strive to obtain, protect, and invest valued resources, and that resource gains can generate gain spirals that facilitate further development (Hobfoll, 2001; Hobfoll et al., 2018). Collaboration with AI may help employees conserve time, energy, and cognitive effort while also providing access to new technological and informational resources. Self-determination theory complements this perspective by explaining how employee-AI collaboration may satisfy employees’ needs for autonomy, competence, and relatedness, thereby activating intrinsic motivation for proactive adaptation and growth (Deci & Ryan, 2000; Ryan & Deci, 2020). Taken together, these two perspectives suggest that employee–AI collaboration can foster career sustainability not only because it provides resources, but also because it motivates employees to invest those resources in proactive work redesign.

This study develops a moderated mediation model in which job crafting mediates the relationship between employee–AI collaboration and career sustainability, and AI job role clarity moderates the relationship between employee–AI collaboration and job crafting. This research seeks to answer three questions by conducting a time-lagged survey: whether employee–AI collaboration predicts career sustainability, how this effect occurs through job crafting, and when this process becomes stronger under conditions of high AI job role clarity. In doing so, this study makes three contributions. First, it advances workplace AI research by moving beyond an adoption-centered perspective and examining employee–AI collaboration as an interdependent work arrangement with implications for long-term career development. This distinction is important because adopting or using AI does not necessarily mean that employees coordinate effectively with AI or derive sustainable career value from it. By focusing on the quality and embeddedness of collaboration, the study provides a more behaviorally grounded explanation of how AI becomes relevant to employees’ careers. Second, this study connects research on AI-enabled work with the career function of job crafting. Prior research has largely treated job crafting as an employee response to technological change or as a predictor of proximal work outcomes. We extend this literature by conceptualizing job crafting as the behavioral conversion mechanism through which employees transform the immediate resource and motivational benefits of AI collaboration into more durable career resources, including adaptability, employability, developmental flexibility, and career renewal. Job crafting therefore explains not only whether employees respond proactively to AI, but also how such responses acquire long-term career value. Third, this study identifies AI job role clarity as a specific coordination boundary condition in employee–AI collaboration. By showing that clear expectations regarding AI functions, human–AI task allocation, and responsibility boundaries strengthen the relationship between employee–AI collaboration and job crafting, the study explains why similar collaborative experiences may generate different behavioral and career consequences. Together, these contributions clarify what is distinctive about employee–AI collaboration, how it may support sustainable careers, and under what conditions this process is more likely to occur. Accordingly, the present study examines employees’ perceived experiences of collaborating with AI and their perceived career sustainability. It does not evaluate the objective technical performance of specific AI systems, nor does it claim that employee–AI collaboration produces uniform effects across technologies, occupations, or industries. Although the time-lagged design temporally separates the measurement of employee–AI collaboration, job crafting, and career sustainability, it does not provide causal identification. The findings should be interpreted as temporal and predictive associations that are consistent with the proposed theoretical framework, rather than as definitive evidence of causal effects.

## 2. Theoretical Background and Hypothesis Development

### 2.1. Conservation of Resources Theory

Conservation of resources theory (COR), originally proposed by Hobfoll (1989), was developed to explain the relationship between stressors and strain and has since been widely applied in organizational research (Demerouti, 2025; Hobfoll et al., 2018; X. Liu & Li, 2025). The theory defines resources as valued objects, conditions, personal characteristics, and energies that support individuals’ survival, functioning, and development (Hobfoll, 2001). Halbesleben et al. (2014) further refined this view by defining resources as anything individuals perceive as helpful for achieving their goals, underscoring the goal-directed and subjective nature of resources. This perspective is particularly relevant to the contemporary AI-enabled workplace, in which employees evaluate whether collaboration with AI helps them perform their work efficiently, conserve effort, and create developmental opportunities (Y. Liu et al., 2025; Sun et al., 2025). In this sense, AI is not merely a technology but part of the organizational resources, shaping how employees perceive, acquire, and deploy valued resources (Call et al., 2026). When AI assists with routine tasks or decision support, it may release employees’ cognitive and psychological resources, thereby creating room for proactive adaptation and long-term career development (Gursoy, 2025; Jarrahi, 2018).

According to COR theory, individuals are motivated to obtain, retain, protect, and cultivate resources, whereas resource loss is experienced as threatening (Hobfoll, 1989). The theory suggests that people must invest resources to prevent further loss, recover depleted resources, and generate new gains (Hobfoll, 2011). This logic offers an insightful explanation for why employee–AI collaboration may predict career sustainability through job crafting. When employees perceive AI as a source of support rather than threat, they tend to use the time, energy, and capability gains derived from AI to proactively redesign their tasks and work boundaries (Qian et al., 2025; Sun et al., 2025). Job crafting can therefore be understood as a form of resource investment through which employees transform immediate resource gains into more durable career resources, such as adaptability, employability, and developmental confidence (Van Wingerden et al., 2017; Zhang et al., 2025). Moreover, COR theory highlights the dynamic nature of resource gain and loss (Hobfoll, 2001; Hobfoll et al., 2018). Employees with greater access to supportive technological resources are more likely to enter positive gain spirals, in which early resource gains from collaboration with AI facilitate proactive behavior and subsequent career benefits (Lin & He, 2026; Shao et al., 2025). By contrast, when resources are insufficient or AI is perceived as threatening, employees may adopt defensive strategies to conserve remaining resources, which may hinder proactive adjustment (He et al., 2024).

### 2.2. Self-Determination Theory

Self-determination theory (SDT) provides another important lens for understanding how employee–AI collaboration may influence career sustainability. This theory argues that human motivation and well-being depend largely on the satisfaction of three basic psychological needs, which are autonomy, competence, and relatedness (Deci & Ryan, 2000; Ryan, 2023). When these needs are supported, individuals are more likely to experience intrinsic motivation, internalize external demands, and engage in proactive and growth-oriented behaviors (Ryan & Deci, 2020). In organizational settings, SDT has been widely used to explain how supportive work environments foster employee initiative, learning, and adaptability (McAnally & Hagger, 2024; Z. Wang & Briand, 2025). In the context of AI-enabled work, collaboration with AI may create conditions that support these basic psychological needs (Chen & Zhao, 2025). For example, AI can help employees handle repetitive tasks and expand solutions, thereby increasing their sense of control over work (Malik et al., 2022). Meanwhile, successful collaboration with AI may strengthen employees’ feelings of competence by enabling them to perform tasks more effectively (Jarrahi, 2018).

From the perspective of SDT, the positive effect of employee–AI collaboration on career sustainability may occur because collaboration with AI activates employees’ internal motivation to adapt, develop, and proactively shape their work (H. Li & Li, 2026). When employees perceive AI as a supportive collaborator rather than a threatening substitute, they are more likely to interpret AI-enabled work as an opportunity for growth and self-development (He et al., 2024). This motivational activation can encourage employees to engage in job crafting, through which they adjust task boundaries, seek new learning opportunities, and align their work more closely with their strengths and career goals (Cheng et al., 2023). Moreover, this perspective helps explain why AI job role clarity is important. AI job role clarity can reduce role ambiguity in AI-enabled work and increases perceived control, which facilitates the translation of employee–AI collaboration into proactive job crafting (Chowdhury et al., 2022). SDT therefore complements COR theory by showing that employee–AI collaboration not only provides resources, but also activates the motivational processes through which those resources are translated into sustainable career development.

### 2.3. Integrating COR Theory and SDT

Although COR theory and SDT emphasize different theoretical mechanisms, they are complementary in explaining how employee–AI collaboration relates to job crafting and career sustainability. COR theory provides the resource-based foundation of the model. From this perspective, employee–AI collaboration represents an important work resource because AI can provide employees with informational support, analytical assistance, task feedback, and problem-solving capacity (Call et al., 2026). These resources may reduce employees’ perceived resource loss and create opportunities for further resource gain (Y. Liu et al., 2025). As a result, employees are more likely to engage in job crafting as a proactive strategy to acquire, protect, and develop valuable work resources.

SDT complements this resource-based explanation by clarifying the motivational process through which such resources are translated into proactive behavior. When employees collaborate effectively with AI, they may experience greater competence because AI helps them perform tasks more efficiently and accurately (Malik et al., 2022). They may also experience greater autonomy when AI enables them to adjust work methods and make more informed decisions (Chen & Zhao, 2025). In addition, effective collaboration may support relatedness when AI use improves coordination with colleagues and enhances employees’ contribution to team goals. These supportive experiences can strengthen employees’ intrinsic motivation to redesign their tasks, relationships, and cognitive boundaries through job crafting.

Therefore, COR theory explains why employee–AI collaboration provides the resources needed for job crafting, whereas SDT explains how these resources activate employees’ self-determined motivation to craft their jobs. Together, the two theories offer a more complete explanation of the proposed model. Employee–AI collaboration creates resource conditions, these conditions support basic psychological needs, and job crafting serves as the behavioral mechanism through which employees transform these resources and motivational experiences into career sustainability.

### 2.4. Employee–AI Collaboration and Career Sustainability

Employee–AI collaboration denotes employees’ ongoing work process of using and coordinating with AI tools to complete job tasks (Sowa et al., 2021). As an indicator of AI embeddedness in organizational routines and employee work practices, this form of collaboration is increasingly linked to enhanced well-being and productivity (Kong et al., 2023). In many work settings, employee–AI collaboration reshapes task allocation between employees and intelligent systems, reducing employees’ involvement in repetitive tasks while increasing their engagement with complex problem solving (Jia et al., 2024). Such changes may help employees conserve valuable resources, including time, energy, and cognitive capacity, while expanding opportunities for learning and skill development (Sun et al., 2025). From the perspective of COR theory, employee–AI collaboration may therefore enhance career sustainability by helping employees preserve existing resources and generate new ones that are relevant for long-term career adaptation.

Furthermore, SDT suggests that individuals are not merely passive recipients of technological change, but are motivated to respond proactively when their needs for autonomy and competence can be supported (Gagné et al., 2022). In AI-enabled work contexts, employees may interpret collaboration with AI as an opportunity for growth rather than merely as a source of disruption (Kunz et al., 2025). By using AI to handle routine work and information processing, employees can redirect their effort and attention toward tasks requiring judgment and creativity (Tschang & Almirall, 2021). This process may strengthen their sense of autonomy, activate feelings of competence, and reinforce intrinsic motivation (Ryan & Deci, 2020). Moreover, successful collaboration with AI can improve employees’ ability to transform inputs into desired outcomes, thereby enhancing work-related flexibility, decision-making capacity, and broader employability (Anthony et al., 2023; McAnally & Hagger, 2024). The digital skills, adaptive experience, and cross-boundary collaboration capabilities developed through working with AI may also become transferable career resources that help employees respond more effectively to future uncertainty and technological change (Jin et al., 2026; Kundi et al., 2024).

Taken together, employee–AI collaboration may predict career sustainability by both activating employees’ internal motivation and facilitating the conservation and accumulation of valuable career resources. Accordingly, we propose the following hypothesis:

**Hypothesis** **1.** *Employee-AI collaboration is positively related to career sustainability.*

### 2.5. Mediating Role of Job Crafting

Job crafting refers to the self-initiated changes employees make to their tasks, relationships, and ways of working to better align their jobs with their abilities, needs, and goals (Tims et al., 2012; Zhang & Parker, 2019). Collaboration with AI often reshapes task allocation, work processes, and skill requirements (Chen & Zhao, 2025). On the one hand, AI can assist with repetitive activities, thereby reducing routine workload and freeing employees from some burdensome tasks (Zhao et al., 2025). On the other hand, it may increase employees’ involvement in more complex and less structured work, which requires them to reconsider how they organize their tasks and approach work goals (Shao et al., 2025). Under such conditions, employees may be more likely to proactively modify their jobs by seeking additional structural resources, taking on more challenging demands, and redefining how work is performed (Liang et al., 2025; Lin & He, 2026).

This tendency can be explained from both SDT and COR theory. From a self-determination perspective, collaboration with AI may support employees’ needs for autonomy and competence (Chen & Zhao, 2025; Ryan & Deci, 2020). As AI takes over part of routine execution and provides analytical or decision support, employees may gain greater discretion in how to complete their work and greater opportunities to develop new skills (Haesevoets et al., 2021). Rather than simply deskilling work, AI can also stimulate reskilling by pushing employees to broaden their capabilities and adapt to new technological demands (Tschang & Almirall, 2021). When employees perceive AI as a supportive collaborator, they may become more motivated to learn and reshape their work in ways that enhance personal growth and effectiveness (He et al., 2024). In this sense, job crafting can be understood as a behavioral response through which employees translate AI-enabled autonomy and competence into proactive work redesign (Song et al., 2022).

From a COR perspective, employee–AI collaboration also creates new opportunities for resource acquisition and investment (Y. Liu et al., 2025). Working with AI may provide employees with technological support, save time and energy, and enhance access to information and problem-solving tools (Call et al., 2026). These gains can enrich employees’ structural, cognitive, and social resources, making them more willing to invest resources in proactive adjustments to their work (Van Wingerden et al., 2017; Zhang et al., 2025). Moreover, as AI increasingly becomes a workplace collaborator rather than a tool, employees may also need to reconfigure their interactions with supervisors and coworkers, coordinate task interdependence, and build new forms of social support around human–AI collaboration (Nazeer & Ahmad, 2025). Such processes can further stimulate relational crafting and the accumulation of social resources (Demerouti, 2025; Rofcanin et al., 2019).

Drawing on COR theory, employee–AI collaboration may provide employees with valuable resources, including task-related knowledge, analytical support, and performance feedback (Zhang et al., 2025). These resources can help employees reduce unnecessary effort and identify new ways to improve their work. From the perspective of SDT, such resource support may also enhance employees’ sense of competence and autonomy, which motivates them to take proactive action in shaping their jobs (Ryan & Deci, 2020). Therefore, employee–AI collaboration is expected to predict job crafting because it not only increases employees’ available work resources but also strengthens their self-determined motivation to use these resources proactively. We propose the following hypothesis:

**Hypothesis** **2.** *Employee–AI collaboration is positively related to job crafting.*

Job crafting may be an important pathway through which employees build and sustain their long-term careers (Akkermans & Tims, 2017). Career sustainability reflects an individual’s capacity to maintain a viable, meaningful, and adaptable career over time across changing work roles and contexts (Chin et al., 2019). In rapidly changing career environments, sustainable careers increasingly depend on employees’ self-directed efforts to align their work with their evolving abilities, values, and developmental goals (De Vos et al., 2020). From this perspective, job crafting is not merely a short-term coping behavior, but a proactive strategy through which employees shape work in ways that strengthen the flexibility, resourcefulness, and regenerative capacity of their careers (Y. Liu et al., 2025; Zhang et al., 2025). By engaging in task, relational, and cognitive crafting, employees may increase their sense of control over their work, strengthen their confidence in handling new challenges, and develop a stronger sense of connection between their daily work and broader organizational or personal goals (Geldenhuys et al., 2021; Zhang & Parker, 2019). According to self-determination theory, these experiences help satisfy employees’ needs for autonomy, competence, and relatedness, thereby fostering intrinsic motivation and encouraging them to take greater ownership of their career development (Gagné et al., 2022). When employees redefine their work in ways that better match their strengths and aspirations, they are more likely to experience their careers as sustainable (Van der Heijden et al., 2020).

The positive role of job crafting can also be explained by the conservation of resources theory. Sustainable careers require the acquisition, protection, and renewal of resources that support long-term employability and adaptation (Barthauer et al., 2020). Job crafting helps employees improve the fit between job characteristics and their personal needs and capabilities, which can enhance person-job fit and job satisfaction (J. Li et al., 2023; Tims et al., 2016). In addition, employees who craft their jobs may gain additional structural and social resources, broaden their knowledge base, and engage in challenging assignments that promote learning, adaptability, and career resilience (Wong et al., 2021). Through this process, job crafting can generate a resource gain spiral in which proactive investments in work redesign produce further developmental opportunities and strengthen the continuity, flexibility, and integration of employees’ career over time (Tomas et al., 2023). Accordingly, we propose the following hypothesis:

**Hypothesis** **3.** *Job crafting is positively related to career sustainability.*

Building on the preceding arguments, job crafting is likely to serve as an important mechanism linking employee–AI collaboration to career sustainability. Collaboration with AI can free employees from some routine demands, provide technological support, and create greater space for learning and adaptation (Anthony et al., 2023). These resource gains and motivational benefits may encourage employees to engage in job crafting by adjusting task boundaries, seeking new challenges, strengthening work relationships, and redefining their jobs in more meaningful and growth-oriented ways (Qian et al., 2025; Shao et al., 2025). In turn, such proactive changes can help employees accumulate valuable career resources, including adaptability, competence, employability, and a stronger sense of career control, thereby enhancing career sustainability (Akkermans & Tims, 2017; Sun et al., 2025). In this sense, job crafting represents the behavioral process through which the resource and motivational advantages generated by employee–AI collaboration are transformed into more sustainable career outcomes. Accordingly, employee–AI collaboration may predict career sustainability not only directly, but also indirectly by stimulating employees to craft their jobs in ways that support their long-term development. Therefore, we propose the following hypothesis:

**Hypothesis** **4.** *Job crafting mediates the positive relationship between employee–AI collaboration and career sustainability.*

### 2.6. Moderating Role of AI Job Role Clarity

AI job role clarity refers to employees’ understanding of work design, role expectations, responsibilities, and task arrangements in a work context where humans and AI collaborate (Chowdhury et al., 2022). It reflects the extent to which employees clearly understand how AI changes their work, how tasks and time should be reallocated, and how their own role fits with broader organizational goals and future AI-related plans (Jarrahi, 2018; Lin & He, 2026). In AI-enabled workplaces, role expectations are often dynamic and multidimensional because employees must continuously adapt to shifting task boundaries, evolving performance standards, and ambiguous responsibility structures while also integrating technical, collaborative, and coordinating functions (Kunz et al., 2025; Malik et al., 2022). Under such conditions, the extent to which employees understand AI may shape whether collaboration with AI is experienced as a manageable developmental opportunity or as a confusing and threatening work demand (Brougham & Haar, 2018; J. Li & Huang, 2020).

Based on self-determination theory, such differences in role clarity are likely to influence whether employee–AI collaboration supports employees’ needs for autonomy and competence (H. Li & Li, 2026; Ryan & Deci, 2020). When AI job role clarity is high, employees are better able to understand the respective responsibilities of humans and AI, the goals of the collaborative work process, and the developmental implications of AI adoption (Zaheer et al., 2025). They are therefore more likely to view AI not as a direct threat of replacement, but as a partner that can support performance improvement and career growth (Tu et al., 2025). Clear role expectations can reduce uncertainty, anxiety, and negative anticipations caused by role ambiguity, allowing employees to use AI more effectively as a work resource and to maintain a stronger sense of control over their work (Yang et al., 2025). As a result, the positive effect of employee–AI collaboration on job crafting is likely to become stronger because employees can more confidently invest resources, seek learning opportunities, and proactively adjust their tasks and work strategies in ways that fit the new work environment.

From a conservation of resources perspective, high AI job role clarity also helps employees avoid unnecessary resource depletion (Lin & He, 2026). When employees clearly understand the boundaries of human–AI collaboration, they do not need to spend excessive time and energy testing role expectations, worrying about accountability for AI-related errors, or coping with uncertainty about performance ownership (Chen & Zhao, 2025; Kunz et al., 2025). Instead, they can convert the efficiency and support provided by AI into usable resources for proactive work redesign (Demerouti, 2025). By contrast, when AI job role clarity is low, employees may become confused about their own role and responsibilities, perceive greater AI anxiety and job insecurity, and hesitate to engage in job crafting (Nazeer & Ahmad, 2025; He et al., 2024). In such situations, the benefits of employee–AI collaboration are less likely to be translated into proactive work behaviors. Accordingly, we propose the following hypothesis:

**Hypothesis** **5.** *AI job role clarity moderates the relationship between employee–AI collaboration and job crafting such that the relationship is stronger when AI job role clarity is high.*

**Figure 1:**
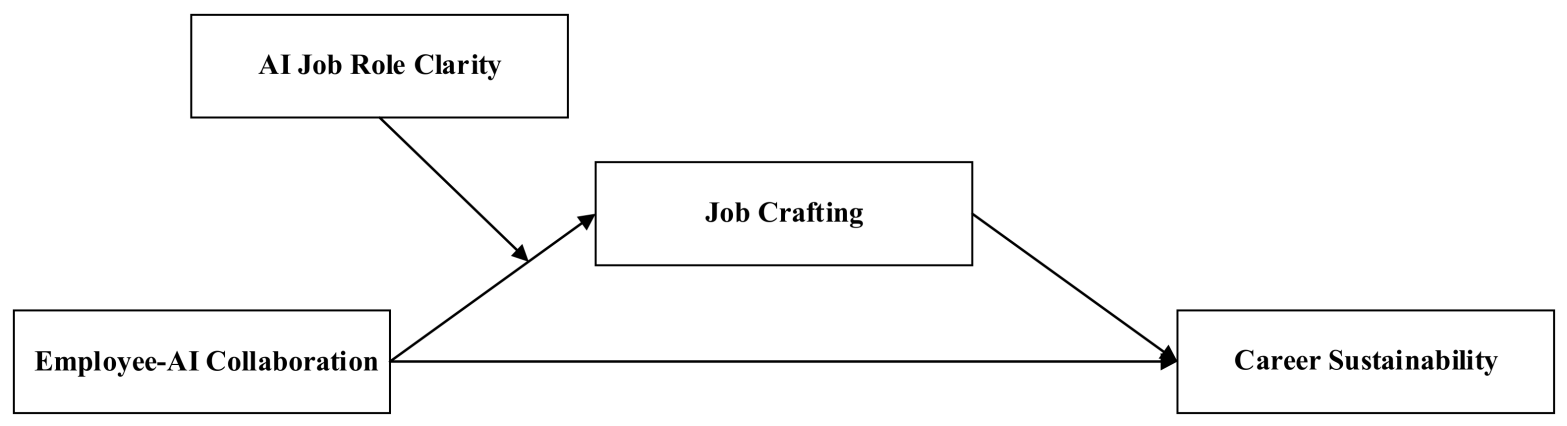
The Theoretical Model.

By combining the above hypotheses, we propose a moderated mediation model as presented in Figure 1, in which AI job role clarity influences the strength of the indirect relationship between employee–AI collaboration and career sustainability through job crafting. As noted above, job crafting is a key pathway through which employees convert immediate work gains into more durable career resources (Akkermans & Tims, 2017). When AI job role clarity is high, employees can more effectively use AI as a supportive resource, identify how their own role should evolve, and proactively adjust their tasks, relationships, and cognitive framing of work (Brougham & Haar, 2018; Chowdhury et al., 2022). These behaviors help them accumulate adaptability, employability, and career confidence, thereby strengthening career sustainability (Blokker et al., 2019; Spurk et al., 2019). In other words, high AI job role clarity makes it more likely that the positive implications of employee–AI collaboration will be translated into sustainable career development through job crafting.

On the contrary, when AI job role clarity is low, employees may be uncertain about task boundaries, responsibility allocation, and the future implications of AI for their work (He et al., 2024; Yang et al., 2025). In this context, collaboration with AI may generate confusion, anxiety, and perceived job insecurity rather than proactive adjustment (Wu et al., 2024). Employees may devote more time and energy to coping with ambiguity and protecting their existing resources, instead of engaging in job crafting (Nazeer & Ahmad, 2025). As a result, the indirect effect of employee–AI collaboration on career sustainability via job crafting is likely to be weaker. In summary, the following hypothesis is proposed:

**Hypothesis** **6.** *The indirect effect of employee–AI collaboration on career sustainability via job crafting is stronger when AI job role clarity is high.*

## 3. Method

### 3.1. Sample and Procedure

We conducted a time-lagged and quantitative survey study. Participants were recruited through Credamo, a professional online panel platform widely used for survey research in China (Zhao et al., 2025). To reduce concerns about common method bias, we used a three-wave data collection design, consistent with Podsakoff et al. (2003). Data collection proceeded across three waves spanning roughly two months. Participants were eligible if they were required to use AI as part of their regular work, irrespective of whether they embraced or resisted such usage. The survey was not designed around a single AI application or employment sector. The original questionnaire recorded respondents’ organizational positions but did not record their industries, occupations, employment arrangements, or the specific AI systems they used. Accordingly, the findings should be interpreted as associations among employees’ perceived AI-enabled work experiences rather than as evidence specific to a particular technology or employment context. Participants were first asked to rate their employee–AI collaboration, AI job role clarity and their demographic information. About three weeks later, they were invited to assess their job crafting. And three weeks later, they were asked to evaluate their career sustainability. Eligible participants were required to be currently employed and to have experience interacting or collaborating with AI technologies in their work. Each participant received 20 RMB as compensation for the time required to complete the multi-wave survey. The payment was not contingent on the content or direction of participants’ responses. Although the platform facilitated access to employees with relevant workplace experience and enabled the matching of responses across survey waves, the use of a single online panel may have introduced self-selection and platform-specific sampling biases.

The use of employee self-reports was consistent with the nature of the focal constructs. Employee–AI collaboration concerns how employees incorporate AI-generated information, recommendations, and task support into their own work processes, whereas AI job role clarity reflects their understanding of the role, responsibilities, and expected contribution of AI. Similarly, job crafting includes self-initiated changes that may not be fully visible to others, and career sustainability captures employees’ perceptions of the adaptability, continuity, and developmental potential of their careers. Employees were therefore considered the most direct source of information regarding these experiences and perceptions. Nevertheless, collecting all focal variables from the same respondents may introduce common method bias. To mitigate, rather than eliminate, this concern, the predictor, mediator, and outcome variables were measured at different time points, with approximately three weeks separating each wave. The questionnaires were also completed anonymously to reduce evaluation apprehension and socially desirable responding.

A total of 523 employees were initially recruited. After excluding 53 incomplete responses and respondents who did not meet the eligibility criteria, 470 valid questionnaires were retained at Wave 1. At Wave 2, 423 valid responses were obtained, and 398 participants subsequently completed Wave 3. Thus, the final sample represented 84.7% of the valid Wave 1 sample, corresponding to a cumulative longitudinal attrition rate of 15.3%. The cases excluded during Wave 1 data screening were not treated as longitudinal attrition because they did not enter the valid baseline sample. In the final sample, 54.5% were men, and 45.5% were women. Among them, 28.1% were aged 21~30, 21.6% were 31~40, 22.4% were 41~50, and 23.6% were 51~60. Concerning educational background, 25.4% had a junior college degree, 46.0% a bachelor’s degree, and 23.6% a master’s degree or above. 78.4% of participants were frontline employees and others were managers. Regarding tenure, 27.1% had worked for less than 3 years, 24.1% for 3~5 years, 21.4% for 5~10 years, and 27.4% for more than 10 years. Table 1 presents the detailed demographic information for final eligible participants in the study.

### 3.2. Measures

For each construct, we identified both the original source of the scale and, where applicable, the subsequent study from which the specific version used in the present research was adopted. Minor wording adjustments were made to align the items with the context of employee–AI collaboration without changing their substantive meanings. Unless otherwise specified, participants rated all items on a five-point Likert scale ranging from 1 (strongly disagree) to 5 (strongly agree). Because the original scales were developed in English, the Chinese questionnaire was prepared using a translation and back-translation procedure to ensure semantic equivalence (Brislin, 1980). The temporal ordering of the measurements is consistent with our theoretical model, but it should not be interpreted as definitive evidence of causality or as excluding possible reciprocal relationships among employee–AI collaboration, job crafting, and career sustainability.

Employee–AI collaboration (α = 0.88). Employee–AI collaboration was defined as the extent to which employees work with AI systems to complete work tasks, obtain task-related support, and integrate AI-generated information or suggestions into their work processes. Employees evaluated their collaboration with AI according to the scale (1 = “strongly disagree”; 5 = “strongly agree”) developed by Kong et al. (2023), which contains five items such as “AI participates in my problems, opportunities, or risk recognition process at work.” The original items were revised to fit the workplace AI context and to capture collaboration between employees and AI systems in daily work. Respondents were asked to indicate the extent to which they agreed with each statement based on their current work experience with AI. Higher scores indicate a higher level of perceived employee–AI collaboration. To improve measurement transparency, the full set of items is reported in Appendix A Table A1. In the present study, employee–AI collaboration was measured at a general perceptual level, referring to employees’ perceived experience of working with AI-enabled systems in performing their job tasks. The measure was not designed to distinguish among specific AI technologies or application categories. Accordingly, the construct reflects respondents’ overall perceptions of employee–AI collaboration rather than technology-specific collaboration experiences.

Job crafting (α = 0.93). Job crafting was assessed using Tims et al.’s (2012) scale (1 = “strongly disagree”; 5 = “strongly agree”), which is one of the most widely used questionnaire for measuring job crafting. The scale captures employees’ self-initiated changes to their job tasks, relationships, or cognitive boundaries. A sample item is “I try to learn new things at work.”

Career sustainability (α = 0.92). Based on Chin et al.’s (2022) twelve-item scale (1 = “strongly disagree”; 5 = “strongly agree”), we asked employees to assess their dimensions of resourceful, flexible, renewable, and integrative. The scale evaluates employees’ perceptions of their capacity to maintain career continuity, development, and adaptability over time. This multidimensional scale includes items like “My career enables me to rebrand or reposition myself”. The scale conceptualizes career sustainability as a multidimensional construct encompassing resourcefulness, flexibility, renewability, and integration, thereby reflecting employees’ perceived capacity to maintain a viable, adaptable, and meaningful career over time. The original validation study provided evidence for the scale’s factorial and nomological validity by examining its theoretically expected relationships with career plateaus, career satisfaction, and psychological well-being.

AI job role clarity (α = 0.93). AI job role clarity was rated using the ten-item scale (1 = “strongly disagree”; 5 = “strongly agree”) developed by Chowdhury et al. (2022). The items assess the extent to which employees clearly understand the role, responsibilities, and expected contribution of AI in their work. A sample item is “I have clarity on why AI systems will be used for specific tasks in my organisation.”

Control variables. We controlled for employees’ gender, age, education, position, as well as tenure in the organization to rule out alternative explanations.

Accordingly, the present measures capture employees’ perceived collaboration with AI, self-initiated job crafting, and perceived career sustainability rather than objective AI usage or externally verified career outcomes. Although employees are appropriate informants for these experiences, reliance on a single respondent source may still inflate associations among the constructs.

## 4. Results

### 4.1. Common Method Bias, Discriminant Validity and Convergent Validity

Given that all focal variables were reported by the same respondents, the observed relationships may be affected by common method bias. We therefore conducted Harman’s single-factor test as an initial diagnostic assessment (Podsakoff et al., 2003). The first unrotated factor accounted for 33.7% of the total variance, below the conventional 50% criterion. We also estimated a confirmatory factor analysis model in which a common latent method factor was specified to load on all measurement items (Widaman, 1985). Compared with the four-factor measurement model, the inclusion of the common latent factor produced only modest changes in model fit (ΔCFI = 0.02, ΔTLI = 0.02, and ΔRMSEA = 0.01). These results suggest that a single common method factor was unlikely to dominate the covariance among the study variables. However, neither Harman’s single-factor test nor the common latent factor analysis can definitively rule out method variance. The results should therefore be interpreted in conjunction with the temporal separation adopted in the research design and the remaining limitation associated with single-source measurement.

Discriminant validity was evaluated through confirmatory factor analyses comparing the fit indices of competing measurement models (Cable & DeRue, 2002). The results in Table 2 suggested the four-factor model had the best fit (χ^2^/df = 1.90 < 3, CFI = 0.93 > 0.9; TLI = 0.92 > 0.9; RMSEA = 0.05 < 0.08; SRMR = 0.04 < 0.08), which indicated good discriminant validity among the study constructs.

Convergent validity was evaluated via composite reliability (CR) and average variance extracted (AVE). Results were as follows: employee–AI collaboration (CR = 0.89, AVE = 0.61), job crafting (CR = 0.94, AVE = 0.51), AI job role clarity (CR = 0.94, AVE = 0.59), and career sustainability (CR = 0.92, AVE = 0.51). All constructs showed CR > 0.80 and AVE > 0.50, providing evidence of satisfactory convergent validity (Cheng et al., 2023).

### 4.2. Descriptive Statistics and Correlations

Table 3 provides an overview of the means, standard deviations, and correlations between the study variables. Employee–AI collaboration was significantly and positively related to career sustainability (r = 0.48, *p* < 0.01), providing preliminary support for Hypothesis 1. Employee–AI collaboration was also significantly and positively correlated with job crafting (r = 0.47, *p* < 0.01), offering initial support for Hypothesis 2. In addition, job crafting was positively correlated with career sustainability (r = 0.58, *p* < 0.01), providing initial support for Hypothesis 3.

### 4.3. Hypotheses Testing

We tested the proposed hypotheses by using hierarchical multiple regression analysis and the results were displayed in Table 4. Model 1 used job crafting as the dependent variable and included control variables as predictors in the regression analysis. In Model 2, employee–AI collaboration was added as the predictor based on Model 1. The hierarchical multiple regression results showed that employee–AI collaboration was positively associated with job crafting (γ = 0.39, *p* < 0.001). Thus, Hypothesis 2 was supported. Model 3 regressed career sustainability on control variables. Employee–AI collaboration was added as an independent variable in Model 4. The results indicated that employee–AI collaboration had a significant positive effect on career sustainability (γ = 0.42, *p* < 0.001). Therefore, Hypothesis 1 was further supported. Building on Model 3, Model 5 included job crafting as an additional predictor. In line with Hypothesis 3, job crafting was positively related to career sustainability (γ = 0.59, *p* < 0.001).

In Model 6, employee–AI collaboration was added to Model 5. The results showed that job crafting remained significantly associated with career sustainability (γ = 0.47, *p* < 0.001), and employee–AI collaboration also had a significant positive effect on career sustainability (γ = 0.23, *p* < 0.001). However, the effect of employee–AI collaboration on career sustainability was weakened after job crafting was included in the model. This finding provided preliminary evidence that job crafting partially mediated the relationship between employee–AI collaboration and career sustainability.

Regarding the control variables, education was positively associated with job crafting in Model 1, whereas age, gender, and organizational tenure were not significantly associated with job crafting. None of the demographic control variables showed a significant association with career sustainability.

To further test the mediating role of job crafting, we conducted a bootstrap analysis with 5000 iterations to obtain 95% confidence intervals (Hayes, 2015). The total effect of employee–AI collaboration on career sustainability through job crafting was 0.42, with a 95% confidence interval of [0.338, 0.487]. The direct effect was 0.23, with a 95% confidence interval of [0.148, 0.318], whereas the indirect effect was 0.18, with a 95% confidence interval of [0.131, 0.238]. Since none of the confidence intervals included zero, the indirect effect of job crafting was significant. These findings provided further support for Hypothesis 4, indicating that job crafting mediated the relationship between employee–AI collaboration and career sustainability.

**Figure 2:**
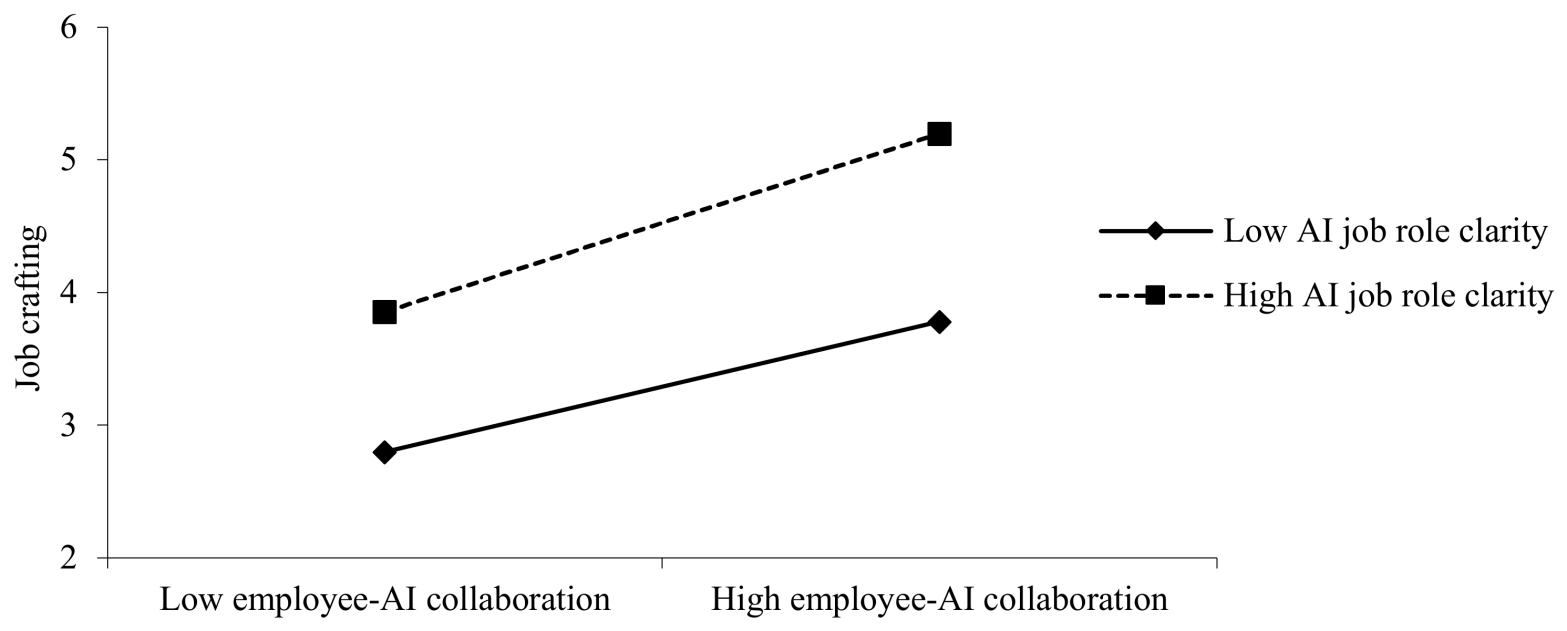
Moderating effect of AI job role clarity in the relationship between employee–AI collaboration and job crafting.

To examine the moderating effect of AI job role clarity, we centered employee–AI collaboration and AI job role clarity before conducting hierarchical multiple regression analysis. The results were displayed in Table 5. Specifically, Model 7 manipulated job crafting as the dependent variable and treated control variables as predictors. Model 8 was developed by entering employee–AI collaboration and AI job role clarity into Model 7. Model 9 was then developed based on Model 8 by adding the interaction term between employee–AI collaboration and AI job role clarity. Consistent with Hypothesis 5, Model 9 revealed that employee–AI collaboration and AI job role clarity jointly and significantly influenced job crafting (γ = 0.10, *p* < 0.05). Figure 2 illustrated the moderating effect of AI job role clarity on the relationship between employee–AI collaboration and job crafting. As shown, the positive relationship between employee–AI collaboration and job crafting was stronger when AI job role clarity was high than when it was low, indicating a positive moderating effect of AI job role clarity.

Following Hayes (2013), we conducted a conditional process analysis to test whether AI job role clarity moderated the mediating effect. Results in Table 6 indicated that, under the condition of high AI job role clarity, the indirect influence of employee–AI collaboration on career sustainability through job crafting remained significantly positive (indirect effect = 0.16, 95% CI = [0.101, 0.221]), but this effect became weaker when AI job role clarity was low (indirect effect = 0.07, 95% CI = [0.019, 0.128]). Moreover, the difference between the two conditions was significant (indirect effect = 0.05, 95% CI = [0.010, 0.087]). Because this confidence interval did not include zero, the indirect effect of employee–AI collaboration on career sustainability through job crafting increased significantly as AI job role clarity increased. Taken together, the moderated mediation effect was significant and Hypothesis 6 was supported.

## 5. Discussion

This study develops a theoretical model to explain how employee–AI collaboration predicts career sustainability through job crafting and how AI job role clarity strengthens this relationship. Drawing on conservation of resources theory and self-determination theory, our research shows that when employees work collaboratively with AI, they are more likely to proactively reshape their jobs, which in turn enhances their career sustainability. Using survey data, we found that employee–AI collaboration was positively associated with career sustainability, that job crafting mediated this relationship, and that AI job role clarity positively moderated the effect of employee–AI collaboration on job crafting. In other words, when employees have a clearer understanding of AI job role, functions and boundaries in the workplace, they are better able to translate collaboration with AI into proactive behavioral adjustments that support their long-term career development. These findings do not imply that AI is uniformly beneficial or that employees should necessarily regard AI as a collaborative partner. Rather, they indicate that, within the present sample, employees who perceived stronger collaboration with AI also reported greater job crafting and career sustainability, particularly when they had a clearer understanding of AI-related roles, functions, and responsibility boundaries.

Nevertheless, these positive associations should not be interpreted as evidence that employee–AI collaboration is uniformly beneficial. Our findings contrast with studies showing that workplace AI can generate job replacement anxiety, learning anxiety, job insecurity, psychological strain, knowledge hiding, and reduced work engagement when employees perceive AI as a threat to their competence, occupational value, or continued employment (Brougham & Haar, 2018; J. Li & Huang, 2020; Y. B. Li et al., 2025; X. Liu & Li, 2025). This apparent inconsistency may reflect the dual resource dynamics of employee–AI collaboration. From a conservation of resources perspective, collaboration with AI can generate informational, temporal, and capability resources, but it can also create resource-loss concerns by increasing learning demands, making existing skills appear obsolete, and introducing uncertainty regarding responsibility and future employment. Similarly, from a self-determination perspective, AI collaboration may support autonomy and competence when AI augments employees’ abilities, but it may frustrate these needs when employees feel displaced, monitored, or unable to meet emerging technological demands. Our results suggest that the resource-gain and need-supporting pathway was more salient on average in the present sample, particularly when employees clearly understood the roles, functions, and boundaries of AI. However, AI job role clarity should be regarded as a facilitating condition rather than a guarantee that negative reactions will not occur. Employees with limited AI competence, high perceived replacement exposure, or insufficient organizational support may still experience collaboration with AI as a source of anxiety and insecurity. Because these negative psychological mechanisms were not directly examined, the present findings do not rule out the possibility that resource-gain and resource-loss processes operate simultaneously. Rather, they indicate that the career consequences of employee–AI collaboration are likely to depend on how employees interpret AI-enabled changes and on whether the surrounding work context enables them to retain control, develop competence, and convert AI-related resources into proactive work adjustments.

The results concerning the control variables also warrant consideration. Education was associated with job crafting in the control variable model, suggesting that employees with greater educational attainment may possess broader knowledge, learning capabilities, or problem-solving resources that facilitate the proactive modification of their work. In contrast, age, gender, and organizational tenure were not significantly associated with job crafting, and none of the demographic controls significantly predicted career sustainability. These findings should not be interpreted as demonstrating that demographic characteristics are generally irrelevant. Instead, they suggest that broad and relatively stable demographic attributes may have limited explanatory power compared with more proximal work experiences and behavioral mechanisms. Employees’ career sustainability may depend less on who they are demographically and more on whether their work environment enables them to acquire resources, exercise autonomy, and proactively adapt their jobs. Moreover, demographic characteristics may influence these outcomes only under occupational or organizational conditions that were not captured in the present model. Future research could therefore examine more theoretically proximal controls, including prior AI experience, digital competence, job complexity, occupational position, and organizational support for AI use.

### 5.1. Theoretical Implications

Our study makes several contributions to the existing literature. First, this study clarifies the incremental contribution of employee–AI collaboration beyond existing research on AI adoption, AI use, and career sustainability. Prior studies have shown that organizational AI adoption, employee AI use, and AI-related appraisals can influence job crafting, proactive behavior, and other employee outcomes (Cheng et al., 2023; He et al., 2024; Y. Liu et al., 2025; Romeo & Lacko, 2026; Schweitzer et al., 2026; Song et al., 2025). Research has also begun to associate employee–AI collaboration with career sustainability, particularly by examining the roles of trust in AI and protean career orientation (Kong et al., 2023; Kunz et al., 2025). We extend this work by placing employee–AI collaboration at the center of the explanatory model and specifying the employee-driven process through which its career implications emerge. Our findings indicate that job crafting enables employees to translate the immediate task, informational, and motivational benefits of collaboration with AI into longer-term career adaptability, renewal, and integration. Thus, the contribution of the present study lies not merely in demonstrating an association between AI and career sustainability, but in explaining how collaborative AI experiences acquire sustainable career value through proactive work redesign.

Second, this study contributes to theory by integrating conservation of resources theory and self-determination theory to explain why employee–AI collaboration can enhance career sustainability. Existing research on AI in the workplace has often relied on a single theoretical perspective, which may not fully capture the complexity of employees’ responses to AI-enabled work contexts (He et al., 2024; Singh et al., 2019; Zhu et al., 2026). By combining these two perspectives, our study develops a more comprehensive explanation of the process through which employee–AI collaboration predicts career sustainability. Specifically, we argue that collaboration with AI can free up employees’ cognitive, temporal and psychological resources while activating intrinsic motivational states by supporting employees’ needs for autonomy, competence and relatedness (H. Li & Li, 2026; Sun et al., 2025). These resource and motivational gains, in turn, encourage employees to engage in job crafting and proactively reshape their work in ways that accumulate long-term career advantages (Kundi et al., 2024; Y. Liu et al., 2025). Thus, our findings suggest a resource and motivation mechanism in which resource release provides the foundation for sustainable career development, whereas motivational activation serves as a key driver translating such resources into proactive behavior and lasting career outcomes. This integrative perspective enriches theorizing on employee adaptation to technological change and helps address the limitations of single theoretical explanations in AI-related contexts.

Third, this study extends research on role clarity by identifying AI job role clarity as a coordination condition specific to human–AI work arrangements. Existing research has mainly examined whether clarity regarding AI roles influences employees’ trust in AI or organizational performance (Chowdhury et al., 2022; Nazeer & Ahmad, 2025). Our findings show that its theoretical relevance extends beyond employees’ attitudes toward AI. AI job role clarity determines whether employees can identify usable opportunities for work redesign within the human–AI division of labor. When employees understand AI functions, task boundaries, responsibility allocation, and performance expectations, they can more confidently invest the resources obtained through AI collaboration in job crafting (Anthony et al., 2023; Kunz et al., 2025; Liang et al., 2025). This finding therefore shifts the role of AI job role clarity from a general cognitive evaluation of AI implementation to a coordination mechanism that facilitates the conversion of employee–AI collaboration into proactive behavior and sustainable career resources.

Finally, although the interaction between employee–AI collaboration and AI job role clarity was statistically significant, its magnitude was relatively modest. This finding indicates that AI job role clarity strengthens, rather than fundamentally transforms, the positive association between employee–AI collaboration and job crafting. From a COR perspective, clear AI-related job responsibilities may reduce uncertainty and conserve the cognitive resources required to determine how collaboration with AI can be translated into proactive job changes. From an SDT perspective, such clarity may also help employees use AI with a greater sense of competence and control. Nevertheless, role clarity is primarily a facilitating contextual resource and cannot substitute for other important antecedents of job crafting, such as job autonomy, AI competence, workload, leadership support, and organizational climate (Chowdhury et al., 2022). Its incremental moderating contribution is therefore likely to be limited once the direct effects of employee–AI collaboration and role clarity are considered. Accordingly, the present finding should not be interpreted as suggesting that role clarification alone will generate substantial changes in job crafting. Rather, it represents one supportive condition that may help employees more effectively convert the resources obtained through AI collaboration into proactive modifications of their work.

### 5.2. Practical Implications

This study provides several practical implications for organizations seeking to use employee–AI collaboration to support proactive adaptation and sustainable career development. First, organizations should move beyond encouraging employees to adopt AI and establish the technical and developmental conditions required for effective collaboration. AI-related training should address not only how to operate systems, but also their capabilities, limitations, appropriate application scenarios, and potential risks. Employees should also be provided with continuing learning opportunities, technical assistance, and access to career development resources so that the immediate benefits of AI collaboration can be converted into transferable knowledge, skills, and longer-term career resources (Jin et al., 2026; Tusquellas et al., 2025; Yin & Hoang, 2026).

More specifically, organizations can enhance AI job role clarity by conducting human–AI task mapping before or during AI implementation. Managers and employees can jointly identify which tasks should be performed by AI, which decisions should remain under human authority, and which activities require collaboration between employees and AI systems (Cheng et al., 2024). These arrangements can be formalized in an AI role charter or similar operating guideline that specifies AI functions, human decision rights, responsibility for AI-assisted outcomes, performance expectations, data-use boundaries, and procedures for escalating uncertain or erroneous AI outputs (Irfan et al., 2023; H. J. Wang et al., 2017). Organizations should subsequently incorporate these expectations into job descriptions, standard operating procedures, performance criteria, onboarding programs, and team briefings. Because AI technologies and work processes continue to evolve, managers should also hold periodic role-review meetings to identify emerging ambiguity and update human–AI responsibilities. Such practices can reduce uncertainty without encouraging employees to rely uncritically on AI.

Organizations should also establish concrete mechanisms that enable employees to engage in job crafting rather than expecting proactive work redesign to occur spontaneously. Managers can organize job-crafting workshops in which employees identify routine tasks that could be supported by AI, higher-value responsibilities they could undertake, new skills they need to develop, and relationships that should be adjusted as human–AI interdependence increases. Regular developmental conversations can be used to translate these ideas into individual action plans. Organizations may further provide protected experimentation time, small-scale pilot projects, peer-learning sessions, and formal channels through which employees can propose changes to task allocation, workflows, and coordination arrangements (Benlian & Pinski, 2025). Supervisors should offer timely feedback and recognize well-designed work improvements, while allowing employees sufficient discretion to adjust how they perform their work. These practices may help employees transform the time, information, and cognitive resources obtained from AI collaboration into task, relational, and cognitive crafting.

However, organizations should not treat role clarification as an isolated or sufficient intervention. The relatively modest moderating effect found in this study suggests that AI job role clarity facilitates job crafting but is unlikely to generate substantial behavioral change by itself. Clear role expectations should therefore be combined with job autonomy, AI competence development, manageable workloads, supportive leadership, and a psychologically safe climate in which employees can experiment with new work arrangements without being penalized for reasonable mistakes. For employees whose roles may be substantially altered by AI, organizations should additionally provide career counseling, reskilling opportunities, internal mobility pathways, job rotation, and participation in cross-functional AI projects (Berg et al., 2010; Zhang et al., 2025). Together, these practices can help employees view AI collaboration as an opportunity to renew their capabilities and career paths while retaining critical judgment and professional autonomy.

### 5.3. Limitations and Future Research Directions

It is important to note that this study has several limitations that warrant further attention. First, all focal variables were measured using employee self-reports. This choice was theoretically consistent with the perceptual and experiential nature of employee–AI collaboration, AI job role clarity, job crafting, and career sustainability, because these constructs may not be fully observable to external raters. Moreover, the three-wave design introduced temporal separation between the predictor, mediator, and outcome variables, and the statistical analyses did not indicate that a single method factor dominated the observed relationships (Avolio et al., 1991). Nevertheless, these procedural and statistical remedies cannot eliminate the possibility that same-source measurement inflated some of the associations. Future research should adopt multi-source and multimethod designs. For example, employees could report their collaborative experiences with AI and perceived career sustainability, supervisors or coworkers could assess observable job-crafting behaviors, AI-system logs could provide behavioral indicators of AI use, and organizational or longitudinal records could capture skill development, internal mobility, employability, or other career outcomes. Such designs would provide a more rigorous assessment of the proposed relationships.

Second, the original survey did not record the specific AI applications used by respondents, their industries or occupations, their employment arrangements, or open-ended explanations for their scale responses. Consequently, we cannot determine whether the observed associations differ across generative AI, algorithmic decision-support systems, robotic systems, or other AI applications, or across occupational and industry contexts. Organizational position was recorded, but it represents hierarchical level rather than occupation or employment type. Because these contextual data were not collected, retrospectively classifying respondents or constructing a table of AI and employment types would not be supported by the original evidence. Future research should prospectively record AI application type, task domain, frequency and intensity of AI use, industry, occupation, and employment arrangement (Anthony et al., 2023; Y. B. Li et al., 2025). Standardized measures could also be supplemented with open-ended questions asking employees to explain their ratings, qualitative interviews, supervisor or coworker assessments, and AI-system usage logs. Such designs would allow researchers to examine whether the relationships identified in this study vary across technologies and employment contexts and would provide stronger triangulation between perceived and behavioral evidence.

Third, the present study primarily examined the resource-gain pathway through which employee–AI collaboration relates to career sustainability. Although we acknowledge that AI may also produce anxiety, job insecurity, learning pressure, and perceived replacement threats, these negative psychological mechanisms were not directly measured in the present study (J. Li & Huang, 2020). Consequently, the positive associations identified here do not indicate that resource-gain and resource-loss processes are mutually exclusive. Employees may simultaneously benefit from the informational and efficiency resources provided by AI while worrying about skill obsolescence, reduced professional autonomy, or future job displacement. Future research could therefore develop dual-path models that examine job crafting and competence development alongside AI anxiety, technostress, and job insecurity. Such research could also investigate whether AI competence, perceived substitutability, organizational support, leadership communication, and AI job role clarity determine which pathway becomes more salient and, ultimately, whether employee–AI collaboration strengthens or undermines career sustainability (Chui et al., 2022; Lamm et al., 2015). Moreover, the external validity of the findings is constrained by the sampling context. All participants were Chinese employees recruited through a single online panel platform. Career norms, employee attitudes toward AI, job crafting practices, and organizational dynamics may vary across cultural and institutional environments. Accordingly, the observed relationships should not be assumed to generalize directly to employees in other countries. Future research could replicate the proposed model using cross-cultural and multi-country samples, assess measurement invariance across cultural groups, and examine cultural values or institutional conditions as potential boundary conditions. In addition, participants received 20 RMB as compensation for completing the multi-wave survey. Although this payment was intended to compensate participants for their time rather than influence their responses, voluntary participation in a compensated online survey may attract individuals who are relatively more engaged in online research or financially motivated. Future studies could reduce this potential self-selection bias by combining multiple recruitment channels, employing organization-based or probability-based sampling, and comparing participants recruited under different incentive arrangements.

Finally, although the time-lagged design introduced temporal separation among the focal variables and was consistent with the proposed theoretical ordering, it cannot definitively establish causal direction. Reverse or reciprocal relationships remain possible. For example, employees who actively craft their jobs may be more likely to seek opportunities to collaborate with AI, while employees who perceive their careers as sustainable may possess greater confidence, adaptability, or willingness to engage with emerging technologies. Employee–AI collaboration, job crafting, and career sustainability may therefore reinforce one another over time rather than operate through a strictly unidirectional process. Future research could employ cross-lagged panel designs with repeated measures of all focal constructs, random-intercept cross-lagged panel models, or experimental interventions that manipulate opportunities for employee–AI collaboration. Such approaches would provide a more rigorous examination of causal direction and reciprocal relationships. Although the study achieved a cumulative retention rate of 84.7% across the three survey waves, participant attrition may still have affected the composition of the final sample. Because systematic information regarding the reasons for withdrawal was not collected, we cannot determine whether participants who discontinued their participation differed from completers in unobserved characteristics. Therefore, potential attrition-related selection bias cannot be completely excluded. Future studies could record reasons for withdrawal and conduct prospective attrition analyses to assess whether continued participation is associated with relevant demographic, occupational, or psychological characteristics.

## 6. Conclusions

Based on conservation of resources theory and self-determination theory, this study examines how employee–AI collaboration is associated with career sustainability. Findings from a multi-wave questionnaire study are consistent with the moderated mediation model proposing that employee–AI collaboration is positively associated with job crafting, which, in turn, is positively linked to career sustainability. Moreover, AI job role clarity strengthens the positive effect of employee–AI collaboration on job crafting. Additionally, the mediating role of job crafting in the relationship between employee–AI collaboration and career sustainability is stronger when AI job role clarity is high. Taken together, the findings offer theoretical and practical insights into the conditions under which employee–AI collaboration is associated with career sustainability.

## Figures and Tables

**Table 1:** Demographic characteristics of the sample.

Variable	Category	Number	Percent
Gender	Male	217	54.5%
	Female	181	45.5%
Age	Below 20	10	2.5%
	21~30	112	28.1%
	31~40	86	21.6%
	41~50	89	22.4%
	51~60	94	23.6%
	Above 60	7	1.8%
Education	Below high school diploma	1	0.2%
	High school diploma	19	4.8%
	Junior college degree	101	25.4%
	Bachelor’s degree	183	46.0%
	Master’s degree or above	94	23.6%
Organizational Position	Frontline employees	312	78.4%
	Junior managers	52	13.1%
	Middle managers	20	5.0%
	Senior managers	14	3.5%
Tenure	Less than 1 year	55	13.8%
	1~3 years	53	13.3%
	3~5 years	96	24.1%
	5~10 years	85	21.4%
	More than 10 years	109	27.4%

Note. *N* = 398.

**Table 2:** Goodness-of-fit indices of the measurement model and alternative models.

Model	χ^2^	df	χ^2^/df	CFI	TLI	RMSEA	SRMR
1. Four-factor model	1548.24	813	1.90	0.93	0.92	0.05	0.04
2. Three-factor model ^a^	2383.37	816	2.92	0.84	0.83	0.07	0.08
3. Two-factor model ^b^	3492.68	818	4.27	0.73	0.71	0.09	0.10
4. One-factor model ^c^	4719.87	819	5.76	0.60	0.58	0.11	0.11

Notes. *N* = 398; ^a^ Combining employee–AI collaboration with AI job role clarity; ^b^ Combining employee–AI collaboration with AI job role clarity, combining job crafting with career sustainability; ^c^ Combining all variables into one factor.

**Table 3:** Means, standard deviations, and correlations between the study variables.

Variable	M	SD	1	2	3
1. Employee–AI collaboration	3.37	0.97			
2. Job crafting	3.42	0.85	0.47 **		
3. AI job role clarity	3.50	0.92	0.42 **	0.49 **	
4. Career sustainability	3.43	0.86	0.48 **	0.58 **	0.48 **

Notes. *N* = 398; ** *p* < 0.01.

**Table 4:** Results of hierarchical multiple regression analysis.

Predictors	Job Crafting		Career Sustainability			
	Model 1	Model 2	Model 3	Model 4	Model 5	Model 6
Constant	2.60	1.56	3.21	2.10	1.68	1.37
Control variables						
Gender	0.10	0.09	−0.07	−0.07	−0.13	−0.12
Age	0.01	0.01	−0.03	−0.04	−0.04	−0.04
Education	0.15 **	0.08	0.13	0.05	0.04	0.01
Position	0.11	0.08	0.03	−0.01	−0.04	−0.04
Tenure	−0.03	−0.01	−0.03	−0.01	−0.02	−0.01
Independent variable						
Employee–AI collaboration		0.39 ***		0.42 ***		0.23 ***
Mediating variable						
Job crafting					0.59 ***	0.47 ***
R^2^	0.04	0.23	0.02	0.24	0.33	0.40
ΔR^2^	0.04	0.20	0.02	0.21	0.32	0.16
F	2.97	19.71 ***	1.70	20.06 ***	34.18 ***	36.79 ***

Notes. *N* = 398; ** *p* < 0.01, *** *p* < 0.001.

**Table 5:** Results of hierarchical multiple regression analysis for moderating effect.

Predictors	Job Crafting		
	Model 7	Model 8	Model 9
Constant	2.60	0.70	0.73
Control variables			
Gender	0.10	0.09	0.09
Age	0.01	0.03	0.02
Education	0.15	0.12	0.11
Position	0.11	0.07	0.06
Tenure	−0.03	−0.03	−0.02
Independent variable			
Employee–AI collaboration		0.25 ***	0.25 ***
Moderating variable			
AI job role clarity		0.34 ***	0.33 ***
Interaction term			
Employee–AI collaboration × AI job role clarity			0.10 *
R^2^	0.04	0.34	0.35
ΔR^2^	0.04	0.31	0.01
F	2.97	28.96	26.33

Notes. *N* = 398; * *p* < 0.05, *** *p* < 0.001.

**Table 6:** Results of the conditional process analysis.

AI Job Role Clarity	Moderator Value	Effect	SE	LLCI	ULCI
Low	M − 1 SD	0.072	0.028	0.019	0.128
Mean	M	0.115	0.023	0.071	0.163
High	M + 1 SD	0.158	0.030	0.101	0.221
Between-group variance	High − Low	0.046	0.020	0.010	0.087

## Data Availability

The data presented in this study are available from the corresponding author upon reasonable request. The data are not publicly available because the informed consent provided to participants did not include the unrestricted public dissemination of individual-level survey responses. In addition, although direct identifiers were removed, the dataset contains demographic, occupational, and longitudinal matching information that may present residual risks of participant re-identification.

## Abbreviations

The following abbreviations are used in this manuscript:

AI	Artificial Intelligence
COR	Conservation of resources
SDT	Self-determination theory
CFI	Comparative Fit Index
TLI	Tucker–Lewis Index
RMSEA	Root Mean Square Error of Approximation
SRMR	Standardized Root Mean Square Residual
CR	Composite Reliability
AVE	Average Variance Extracted

## Appendix A

**Table A1:** Measurement of employee–AI collaboration.

Item	Cronbach’s α if Item Deleted	Standardized Factor Loading
AI participates in my decision-making process at work.	0.853	0.781
AI participates in my prediction process at work.	0.851	0.781
AI participates in my problem-solving process at work.	0.851	0.777
AI participates in my information identification and evaluation process at work.	0.850	0.773
AI participates in my problems, opportunities, or risk recognition process at work.	0.851	0.790
